# Supplementary material for: Treatment Realities of Headache Disorders in Rural Germany by the Example of the Region of Western Pomerania
Source: Brain Sci. 2021 Jun 24;11(7):839. doi: 10.3390/brainsci11070839 (PMC8301947; doi:10.3390/brainsci11070839)
Supplement: Supplementary file 1 [file brainsci-11-00839-s001.zip › brainsci-1243268-supplementary.pdf]

How often do you treat patients with headaches as their main diagnosis in your medical practice?

☐ Daily ☐ Several times a week ☐ Several times a month ☐ Less than 1x/month

What applies the best concerning your treatment of the following headache diagnoses?

	I always treat by myself…	I often treat by myself…	I sometimes treat by myself….	I always refer the patient.
Migraine	☐	☐	☐	☐
Tension Headache	☐	☐	☐	☐
Symptomatic headache (e.g. after a trauma, …)	☐	☐	☐	☐
Other	☐	☐	☐	☐
Diagnosis unknown	☐	☐	☐	☐

For what reasons do you refer headache patients? (more than one selection possible)

☐ Diagnosis not possible
☐ Treatment attempts not successful
☐ No further treatment options
☐ No treatment according to guidelines possible
☐ Generally with certain diagnoses
☐ Patient wishes for a referral
☐ Other:

To which specialist do you refer patients? (more than one selection possible)

☐ Neurologist
☐ Orthopaedist
☐ Pain therapist
☐ Headache specialist (independent from specialisation) or headache outpatient clinic
☐ Other specialisation:

Do you know local specialized headache therapists or headache outpatient clinics?

☐ Yes, a registered headache specialist

Yes, a headache outpatient clinic

No

Would you appreciate further advanced training possibilities concerning the treatment and diagnosis of headache disorders?

☐ Yes ☐ No, there are sufficient possibilities

Supplementary Table S1 – Questionnaire for general practitioners concerning their treatment of headache patients.
